# Transcranial focused ultrasound phase correction using the hybrid angular spectrum method

**DOI:** 10.1038/s41598-021-85535-5

**Published:** 2021-03-22

**Authors:** Steven A. Leung, David Moore, Taylor D. Webb, John Snell, Pejman Ghanouni, Kim Butts Pauly

**Affiliations:** 1 Department of Bioengineering, Stanford University, Stanford, CA USA; 2 Focused Ultrasound Foundation, Charlottesville, VA USA; 3 Department of Electrical Engineering, Stanford University, Stanford, CA USA; 4 Department of Neurological Surgery, University of Virginia, Charlottesville, VA USA; 5 Department of Radiology, Stanford University, Stanford, CA USA

**Keywords:** Biomedical engineering, Medical imaging

## Abstract

The InSightec Exablate system is the standard of care used for transcranial focused ultrasound ablation treatments in the United States. The system calculates phase corrections that account for aberrations caused by the human skull. This work investigates whether skull aberration correction can be improved by comparing the standard of care InSightec ray tracing method with the hybrid angular spectrum (HAS) method and the gold standard hydrophone method. Three degassed ex vivo human skulls were sonicated with a 670 kHz hemispherical phased array transducer (InSightec Exablate 4000). Phase corrections were calculated using four different methods (straight ray tracing, InSightec ray tracing, HAS, and hydrophone) and were used to drive the transducer. 3D raster scans of the beam profiles were acquired using a hydrophone mounted on a 3-axis positioner system. Focal spots were evaluated using six metrics: pressure at the target, peak pressure, intensity at the target, peak intensity, positioning error, and focal spot volume. For three skulls, the InSightec ray tracing method achieved 52 ± 21% normalized target intensity (normalized to hydrophone), 76 ± 17% normalized peak intensity, and 0.72 ± 0.47 mm positioning error. The HAS method achieved 74 ± 9% normalized target intensity, 81 ± 9% normalized peak intensity, and 0.35 ± 0.09 mm positioning error. The InSightec-to-HAS improvement in focal spot targeting provides promise in improving treatment outcomes. These improvements to skull aberration correction are also highly relevant for the applications of focused ultrasound neuromodulation and blood brain barrier opening, which are currently being translated for human use.

## Introduction

Transcranial focused ultrasound is a therapeutic modality that can be used to non-invasively induce thermal or biomechanical effects in the brain. Ultrasound is focused through the skull to achieve transient or permanent changes in brain tissue, and the durability of these changes depend on the parameters used during treatment. The most common applications include ablation^1–4^ as an alternative to open brain surgery, neuromodulation^5–7^ to probe the circuitry of the brain, and blood brain barrier opening^8–11^ to improve drug delivery into the brain.

**Figure 1:**
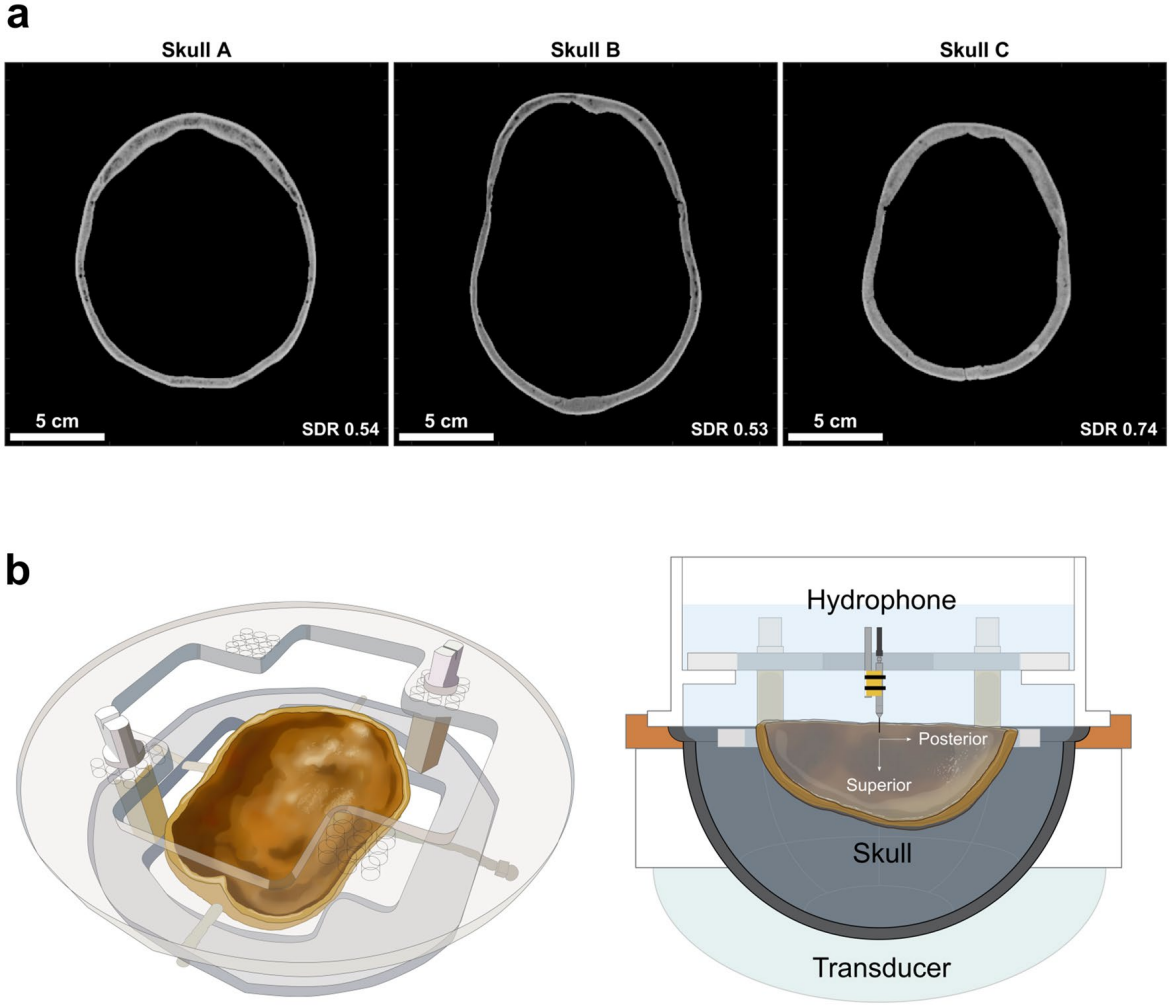
Skulls and experimental setup used in this study. (**a**) Three ex vivo human skulls were used for experimentation. They varied in size, shape, thickness, and bone composition. Their scale bars and skull density ratios (SDRs) are shown at the bottom of each image. (**b**) Each skull was fixed to a head frame to ensure consistent positioning. The head frame was secured in an InSightec 670 kHz hemispherical phased array transducer. Data were acquired with a needle hydrophone and 3-axis positioner system. Illustrations were drawn by Sarah Hwang.

The intact skull poses a major technical challenge for transcranial focused ultrasound. It defocuses the ultrasound focal spot and shifts the focal spot away from the target location. The complexity of this defocusing is largely due to the heterogeneous nature of the skull. Within one skull, there is variability in shape, thickness, and bone composition. Thus, two separate locations on the same skull can experience substantial differences in ultrasound propagation speed, reflection, refraction, and attenuation. The interaction of these effects is often too complex to analyze analytically. In addition, skulls are highly heterogeneous across patients, contributing to additional complexity (Fig. 1a).

To improve focusing through the skull, there are several methods that may be used. These methods estimate the aberrating effects of the skull, which can then be accounted for by applying phase corrections to transducer elements. The gold standard for achieving ideal phase corrections is to use a hydrophone-based method^12,13^. A hydrophone is placed at the target location to measure the signals transmitted through the skull. These signals provide exact knowledge of how the skull is affecting ultrasound propagation. The appropriate phase corrections may then be applied to restore constructive interference at the target. Unfortunately, this method requires the placement of a hydrophone inside the brain; its invasiveness is non-ideal. In contrast, the current standard of care is to use an image-based ray tracing method^13,14^. Rays are traced from each transducer element to the target location, and the skull profiles along these rays are used to calculate phase corrections. Ray tracing is a straightforward and intuitive method, but it assumes plane wave propagation of ultrasound and can result in targeting errors. To compensate for these errors, a multi-sonication process is used to apply adjustments in an iterative manner. For transcranial ablation treatments, this iterative process can be achieved by generating small temperature rises and measuring them with magnetic resonance proton resonance frequency shift thermometry (MR thermometry)^15^. However, in applications such as neuromodulation and blood brain barrier opening, the magnitude of temperature rise does not exceed the noise floor of MR thermometry. Thus, either a different mode of measurement is needed for calibration, or better targeting is needed.

Prior studies have proposed the use of simulation methods to bridge the gap in performance between the ray tracing and hydrophone methods^16–18^. However, no study to date has benchmarked against both the standard of care InSightec ray tracing method and the gold standard hydrophone method; a direct comparison to both methods is needed to determine a proposed simulation method’s clinical relevance. Most commonly, simulation studies use finite difference time domain (FDTD)^16,18–28^ or pseudo-spectral time domain (PSTD)^29–33^ methods to simulate the propagation of ultrasound through heterogeneous media. These image-based simulation methods often require many hours of computation time to converge, though recent work has greatly reduced the computation time needed to estimate phase corrections by reducing the computation volume^33^. In contrast to these methods, the hybrid angular spectrum (HAS) method^17,34–38^ requires substantially less computation time when simulating a computation volume of the same size, often running 100×–1000× faster than the FDTD and PSTD methods^36^. This reduction in computation time opens the opportunity for using the HAS method in real-time during transcranial ultrasound treatments. The purpose of this work was to evaluate the performance of the HAS method for phase correction and to compare it against the standard of care InSightec ray tracing method, the gold standard hydrophone method, and a straight ray tracing method used to approximate the proprietary InSightec ray tracing method.

In this study, we performed experiments on three ex vivo human skulls and corrected for the skull aberrations using five sets of phase corrections (no correction, straight ray tracing, InSightec ray tracing, HAS, and hydrophone). We performed 3D hydrophone raster scans with a 5 × 5 × 5 mm field of view to characterize the resulting focal spots and evaluated them using six metrics: pressure at the target, peak pressure, intensity at the target, peak intensity, positioning error, and focal spot volume. Additionally, we investigated sources of error to explore opportunities for future improvement in phase correction estimation.

## Results

### 3D raster scans of phase corrected focal spots

**Figure 2:**
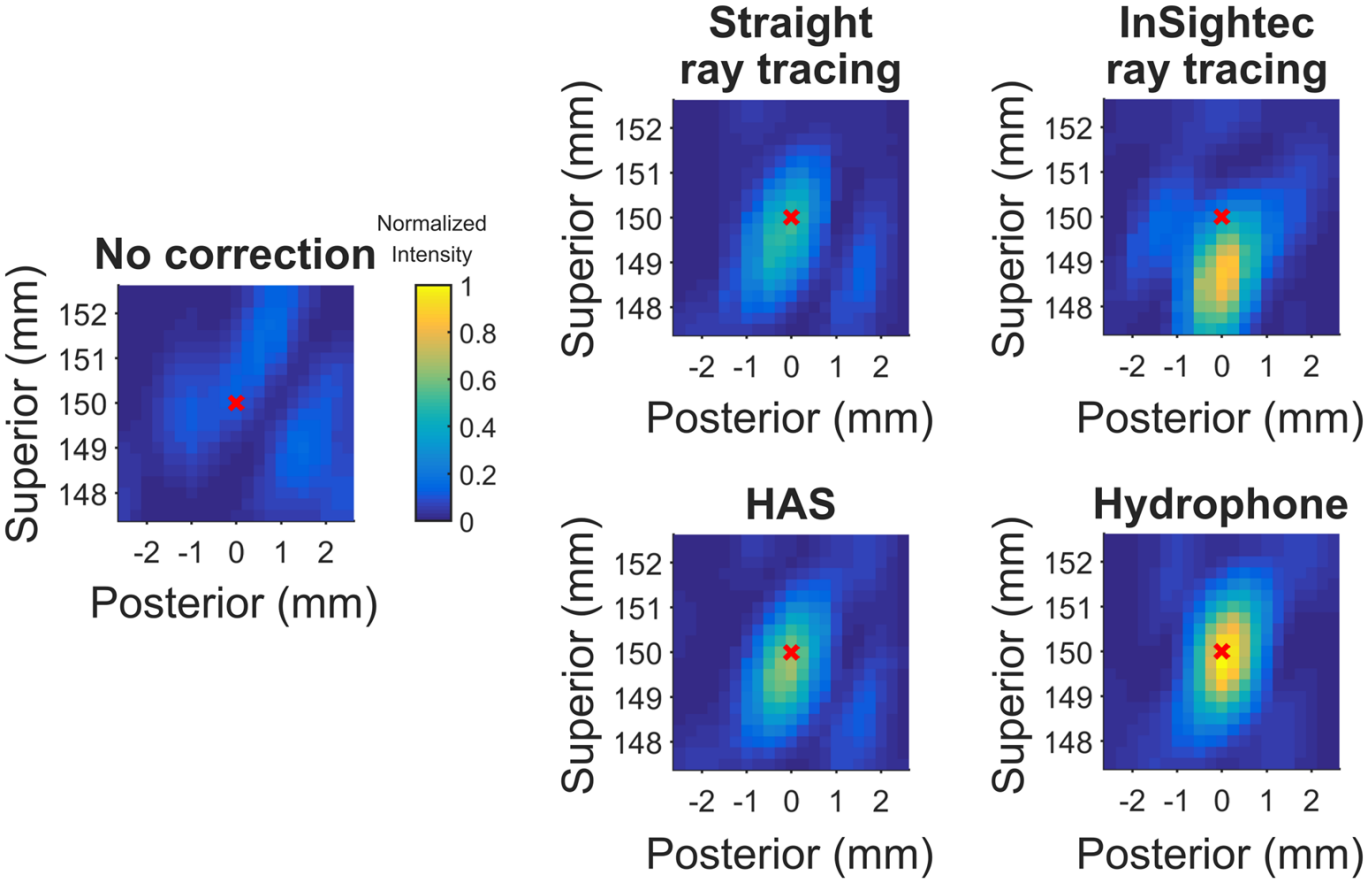
2D cross section raster scans of the refocused focal spot (skull C). The sagittal view is shown. These cross sections are a subset of the 3D volume scans acquired. A red x marks the position of the target, which was placed at the geometric focus.

**Figure 3:**
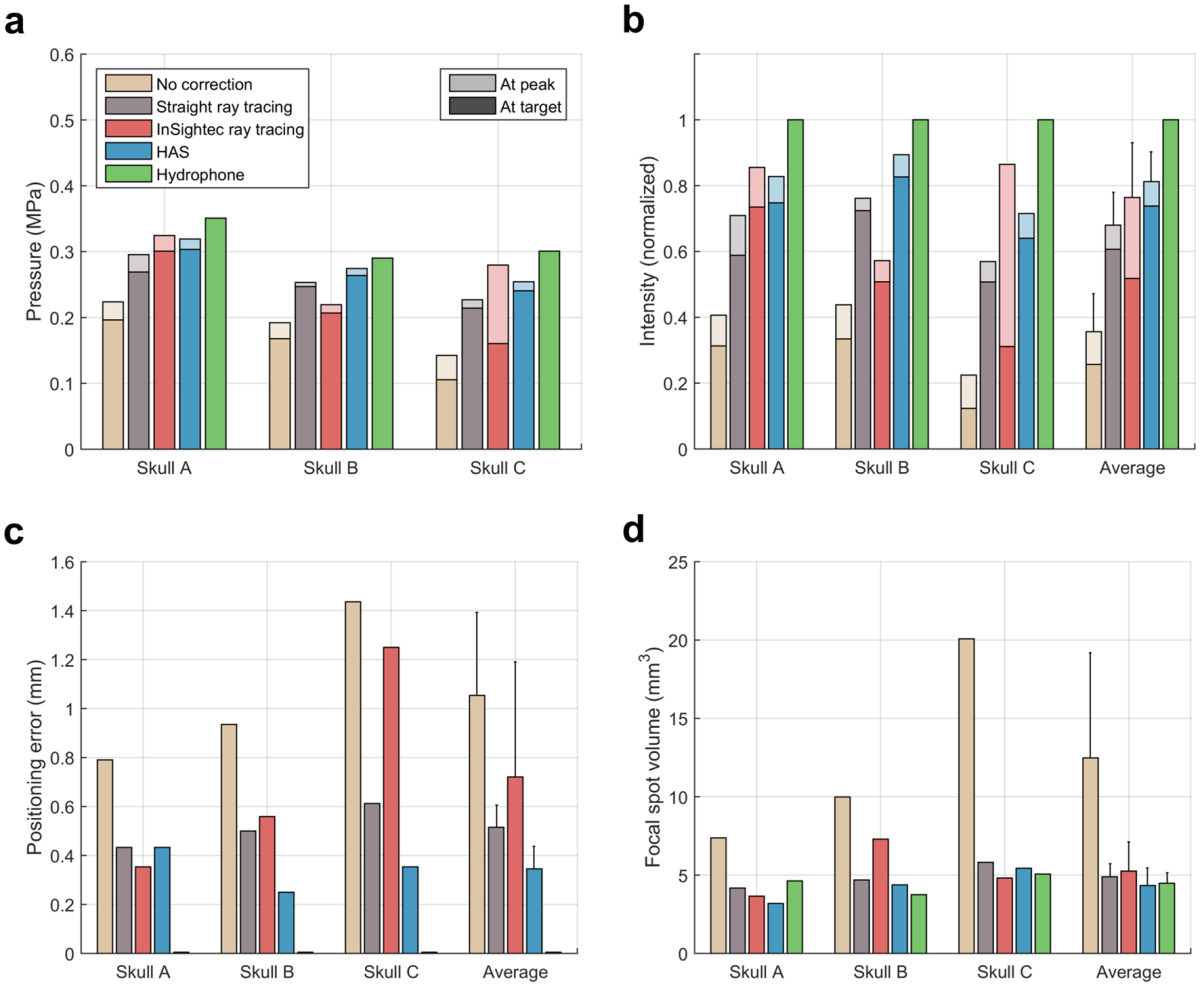
Quantitative metrics used to evaluate the phase correction methods. (**a**) Target and peak pressure (shown with darker and lighter colors, respectively) when operating the transducer at 20 W of electrical power. (**b**) Target and peak intensity normalized to the hydrophone method. (**c**) Focal spot positioning error. (**d**) Focal spot volume. Standard deviation bars are shown.

3D hydrophone raster scans were acquired for five sets of phase corrections: no correction, straight ray tracing, InSightec ray tracing, HAS, and hydrophone. Example normalized intensity images from skull C are shown in Fig. 2. Three-plane cross sections of the focal spots can be found in Supplementary Figures 1, 2, and 3. With no phase correction, the focal spot was defocused and displaced from the geometric focus. The phase correction methods were able to recover the focal spot at or near the geometric focus, with varying pressures and positions. Using the hydrophone method for comparison, the presence of skull reduced transmitted pressure to 25 ± 3% compared to free field. The focal spots were evaluated using six metrics, which are shown in Fig. 3 and summarized in Table 1.

**Table 1:** Summary of results for the metrics used to evaluate phase corrected focal spots.

	No correction	Straight ray tracing	InSightec ray tracing	HAS	Hydrophone
Target pressure	0.16 ± 0.05 MPa	0.24 ± 0.03 MPa	0.23 ± 0.07 MPa	0.27 ± 0.03 MPa	0.31 ± 0.03 MPa
Peak pressure	0.19 ± 0.04 MPa	0.26 ± 0.03 MPa	0.27 ± 0.05 MPa	0.28 ± 0.03 MPa	0.31 ± 0.03 MPa
Target pressure (normalized to hydrophone)	50 ± 13%	78 ± 7%	71 ± 15%	86 ± 5%	100 ± 0%
Peak pressure (normalized to hydrophone)	59 ± 10%	82 ± 6%	87 ± 10%	90 ± 5%	100 ± 0%
Target intensity (normalized to hydrophone)	26 ± 12%	61 ± 11%	52 ± 21%	74 ± 9%	100 ± 0%
Peak intensity (normalized to hydrophone)	36 ± 12%	68 ± 10%	76 ± 17%	81 ± 9%	100 ± 0%
Positioning error	1.05 ± 0.34 mm	0.52 ± 0.09 mm	0.72 ± 0.47 mm	0.35 ± 0.09 mm	0.00 ± 0.00 mm
Focal spot volume	12.48 ± 6.71 mm^3^	4.89 ± 0.84 mm^3^	5.26 ± 1.86 mm^3^	4.33 ± 1.13 mm^3^	4.48 ± 0.67 mm^3^

Compared to the two ray tracing methods, the HAS method resulted in higher target and peak focal spot intensities, less positioning error, and lower focal spot volume. For the straight ray tracing, InSightec ray tracing, and HAS methods, the normalized target intensities were 61 ± 11%, 52 ± 21%, and 74 ± 9%, respectively, and the normalized peak intensities were 68 ± 10%, 76 ± 17%, and 81 ± 9%, respectively (Fig. 3b). Positioning errors were 0.52 ± 0.09 mm, 0.72 ± 0.47 mm, and 0.35 ± 0.09 mm (Fig. 3c), and focal spot volumes were 4.89 ± 0.84 mm^3^, 5.26 ± 1.86 mm^3^, and 4.33 ± 1.13 mm^3^, respectively (Fig. 3d). Although no statistical difference between methods was observed, the HAS method was generally more consistent than the standard of care InSightec ray tracing method, as seen by the standard deviation of the results.

We also quantified focal spot improvements when benchmarked against the no correction case. Compared to no correction, the InSightec ray tracing method achieved 113 ± 54% more target intensity (Fig. 3b), 142 ± 130% more peak intensity (Fig. 3b), and 0.33 ± 0.13 mm less positioning error (Fig. 3c). In comparison, the HAS method achieved 235 ± 160% more target intensity, 142 ± 66% more peak intensity, and 0.71 ± 0.36 mm less positioning error. Overall, compared to target intensity and positioning error for the no correction case, the HAS method achieved approximately twice the amount of improvement than the InSightec ray tracing method.

### Analysis of phase correction efficacy

**Figure 4:**
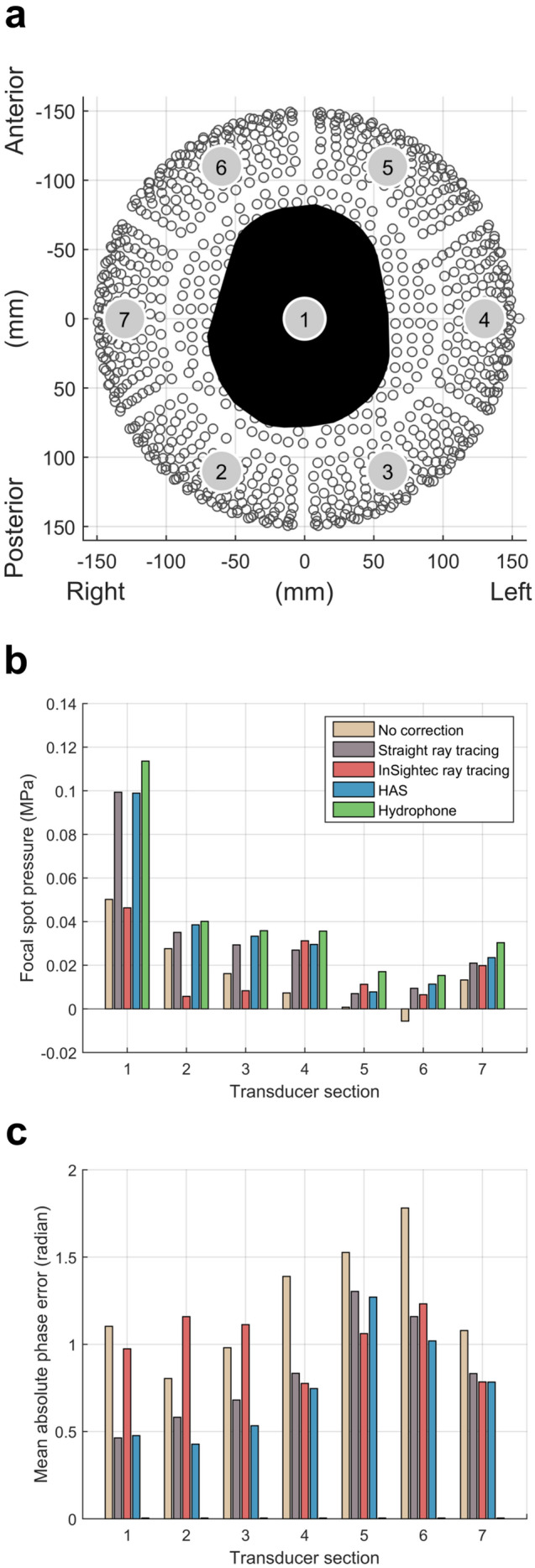
Analysis of phase correction efficacy for each transducer section (Skull C). (**a**) Top-down view looking into the InSightec hemispherical head transducer. It is physically subdivided into seven sections. Radiological left-posterior-superior (LPS) coordinates are shown. (**b**) Contributions by each transducer section to target pressure, calculated with Eq. (5). Negative values denote transducer sections whose net ultrasound signal was out of phase with the rest of the transducer. (**c**) Mean absolute phase errors for each transducer section.

To localize sources of error in phase correction, subsets of elements were analyzed to determine their contributions to the focal spot pressure at the target. Because the InSightec hemispherical head transducer is physically divided into seven sections (Fig. 4a), we used this as one approach for grouping subsets of elements. The bar graphs in Fig. 4b denote the amount of pressure each transducer section contributed to the focal spot pressure at the target, for skull C. The pressure contributions were calculated with Eq. (5) and denote the decrease in target pressure if that subset of elements were turned off. A negative value signifies that the net ultrasound signal coming from the subset was out of phase with the rest of the transducer; turning off those elements would result in a net increase in target pressure. For each section, its contribution to target pressure was substantially higher with phase corrections than with no phase corrections. This was consistent across all skulls. Some skull aberrations were severe enough to cause an entire section to interfere destructively with the other sections, as seen with section 6. Overall, across three skulls, it was more efficient to transmit ultrasound through the superior portion of the skull than through the other portions of the skull, as seen with the hydrophone-corrected pressure. Although section 1 contains exactly twice the number of elements as each of the other sections, the hydrophone-corrected pressure is more than two times as large (Fig. 4b). Furthermore, sections 5 and 6, corresponding to the anterior skull, contributed the least amount of pressure to the target (less than 60% of hydrophone-corrected pressure compared to other sections) and contained the largest mean absolute phase errors (Fig. 4b,c).

For skull C, the InSightec ray tracing focal spot was observed to be displaced 1.25 mm inferior from the target (Figs. 2, 3b,c). This result was consistent with our clinical experience at Stanford with essential tremor treatments: for patients with high SDR (0.7 +), initial alignment sonications can often demonstrate focal heating inferior to the focal target (Supplementary Figure 7). This is one of the reasons why the InSightec clinical protocol necessitates a focal spot alignment stage: to account for initial focal spot positioning error. The focal spot displacement for skull C was unlikely caused by registration error, as shown by the accuracy of the registration method used in this study. Furthermore, the straight ray tracing, InSightec ray tracing, and HAS methods all used the same image registration, yet no systematic displacement of the focal spot was observed. To contextualize the results shown in Fig. 4, the skull C focal spot was not at the target due to the 1.25 mm displacement, therefore the transducer sections did not make large contributions to the pressure at the target. Thus, the InSightec ray tracing method displayed low pressure contributions and high mean absolute phase errors for sections 1, 2, and 3 in addition to sections 5 and 6.

**Figure 5:**
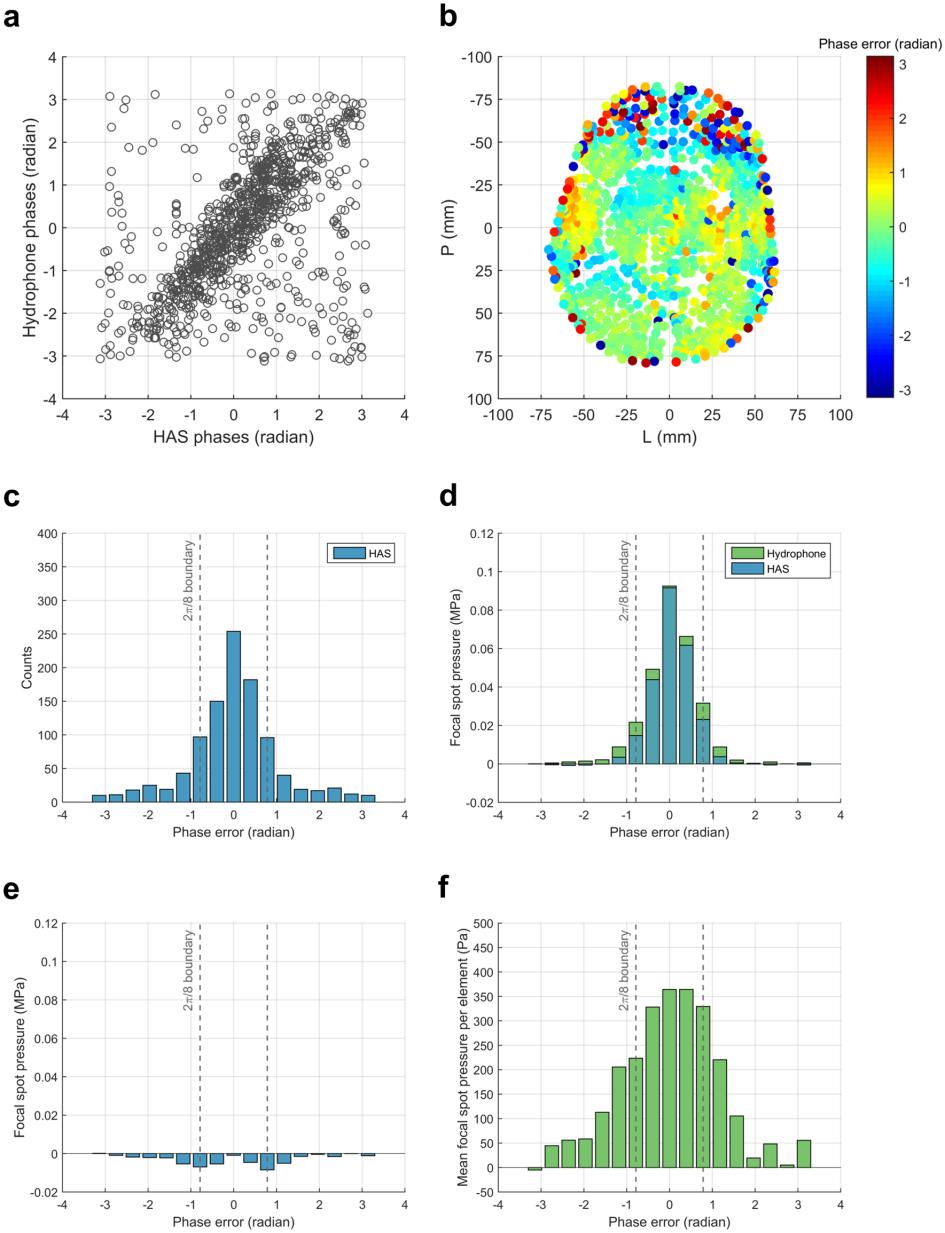
Analysis of phase correction efficacy for subsets of elements grouped by HAS phase error (Skull C). (**a**) Ground truth hydrophone phases plotted against HAS phases. (**b**) HAS phase errors projected onto the surface of the skull. The anterior portion of the skull was associated with larger phase errors. (**c**) Histogram of HAS phase errors. The vertical dashed gray lines denote the 2π/8 boundary condition needed for optimal constructive interference^16^. The phase error bins were used for grouping subsets of elements. (**d**) Each subset’s contribution to target pressure, calculated with Eq. (5). Analysis for the HAS and hydrophone methods are shown. (**e**) Amount of pressure lost due to phase error, calculated by subtraction of hydrophone from HAS using values from (**d**). (**f**) Mean pressure contributed per element, calculated by hydrophone values from (**d**) divided by histogram counts from (**c**).

To further understand how phase errors contributed to changes at the focal spot, additional analysis was performed for the HAS method. The HAS phase error was calculated by taking the difference between the HAS phase corrections and the ground truth hydrophone phase corrections (Fig. 5a). Projecting these phase errors onto the surface of the skull shows that most of the large phase errors were associated with the anterior skull and excision-induced edges of the skull (Fig. 5b). This was in agreement with the transducer section-wise analysis (Fig. 4b). A histogram of the HAS phase error (Fig. 5c) shows that 70% of elements had phase error less than 2π/8, a condition needed for optimal constructive interference^16^. The histogram’s phase error bins were used as a second approach for grouping subsets of elements, for which Fig. 5b can be used to visualize these subsets. The bar graphs in Fig. 5d denote the amount of pressure each phase bin subset contributed to the final focal spot pressure for skull C. Only the analysis for the HAS and hydrophone methods is shown. For each phase bin subset, the elements contributed more pressure to the target when using hydrophone corrections than when using HAS corrections. The difference between the two methods shows that most of the unrecovered pressure is due to phase errors in the ± [0.59 to 1.37] radian range (Fig. 5e). Although 30% of elements had HAS phase errors greater than 2π/8, those elements contributed 6% of focal spot pressure at the target. By removing those phase errors, the same elements would contribute a total of 14% of focal spot pressure. Seeing that these elements contributed less focal spot pressure than the number of elements would suggest, we investigated whether this could be a consequence of ultrasound transmission through the skull. The mean pressure per element (Fig. 5f) was calculated by dividing Fig. 5d’s hydrophone pressures by the HAS histogram counts in Fig. 5c. On average, elements with larger HAS phase error were associated with lower pressure transmission through the skull. Together, Fig. 5e,f show that larger phase errors may not necessarily be more problematic than smaller phase errors, because phase errors are less destructive when paired with small pressure amplitudes.

## Discussion

In this study, we directly compared several phase correction methods in their ability to refocus the focal spot at the intended target. A single focal spot was generated using each method to emulate how an initial sonication would perform; no focal spot calibration was performed. Compared to the two ray tracing methods, the HAS method achieved higher pressure and intensity at the target, less positioning error, and less focal spot volume. Although large phase errors were observed, they did not substantially affect focal spot pressure and intensity. With the HAS method’s ability to accurately refocus the focal spot, it has great potential to be used for treatment planning in applications such as ablation, neuromodulation, and blood brain barrier opening.

Compared to the two ray tracing methods, the HAS method achieved higher target and peak focal spot intensities and less positioning error, all of which are highly relevant to the clinical setting. During transcranial ablation treatments, a multi-sonication process is often needed to adjust for targeting errors. This is a time-consuming process in which after each sonication, the surgical team assesses patient symptoms and behavioral readouts to ensure patient safety. To confirm targeting, there is no defined limit to the number of rounds of sonication and patient assessment needed. For essential tremor treatments where the anatomical target is small, this process typically does not need to be repeated extensively. However, for future ablation applications such as glioblastoma or brain metastases, multiple targets or larger volumes of treatment may be needed, necessitating multiple rounds of iterative target calibration and patient assessment. Thus, reducing targeting error from the initial focal spot could streamline the clinical workflow and substantially reduce treatment duration. Furthermore, with consistent performance across different skulls and improvements over the standard of care InSightec ray tracing method, the HAS method may enable improved patient outcomes both in terms of efficacy and consistency. It may also enable a larger subset of the patient population to receive treatment, and thus indirectly influence the patient screening process.

For applications such as neuromodulation and blood brain barrier opening, improvements in targeting are also clinically relevant. Due to negligible temperature rise at the focal spot, the use of MR thermometry for target calibration is not feasible. Therefore, other monitoring and feedback techniques are needed instead. MR acoustic radiation force imaging (MR ARFI) is one such alternative for determining focal spot position and intensity, however, it is not currently utilized during treatments due to low signal to noise ratio when used with the InSightec Exablate transducer. Without reliable focal spot monitoring and target calibration, the feasibility of clinical treatments will be limited by the accuracy of the phase correction methods. Based on results from this study, the InSightec ray tracing and HAS methods had focal spot positioning errors reaching up to 1.25 mm and 0.43 mm, respectively (Fig. 3c). For context, the InSightec transducer has a nominal focal spot size of 1.5 × 1.5 × 2.5 mm at pressure full width half maximum (Supplementary Figure 8). A positioning error of 0.75 mm in the transverse plane or 1.25 mm in the axial plane is enough to substantially reduce the amount of ultrasound energy delivered to the target. At the same time, positioning error would substantially increase the amount of undesired energy delivered to other regions within the brain. With the maximum positioning errors of 1.25 mm and 0.43 mm, the pressures at the target were reduced by 43% and 5% for the InSightec ray tracing and HAS methods, respectively. Although the HAS method was shown to consistently deliver ultrasound energy to the desired target, it is worth additional research and development to further reduce positioning error and improve maximum pressure recovery.

Although FDTD and PSTD methods have been used in prior studies to investigate transcranial phase corrections with phased array transducers, this study is the first to use the HAS method with a hemispherical phased array transducer, and the first to compare it against the standard of care InSightec ray tracing method and the gold standard hydrophone method. To contextualize the HAS method’s performance against full-wave FDTD and PSTD methods, we can consider the results from Marsac et al.^18^ and Maimbourg et al.^33^. However, as a caveat, comparison of results across studies are often complicated by differences in experimental setup, including but not limited to skull samples, transducer frequency, and transducer f-number. The skulls used for experimentation are an especially important consideration, because there exists a fourfold range of ultrasound transmission efficiencies through the skull^35^. Marsac et al. used an FDTD method for phase correction, whereas Maimbourg et al. used a PSTD method for phase correction. With the skull in place and with no phase correction, the normalized focal spot peak pressures were 45 ± 3% and 61 ± 11% for Marsac et al. and Maimbourg et al., respectively. Following phase correction, the normalized peak pressures were 86 ± 3% and 90 ± 3%, respectively. These peak pressures were comparable with the 90 ± 5% normalized peak pressure from this study. The average positioning error was also comparable, with average errors of 0.54 mm, 0.60 mm, and 0.35 mm for Marsac et al., Maimbourg et al., and this study, respectively. The fourfold range in skull transmission efficiencies was not observed across these three studies, though it is unclear whether this is because these sophisticated phase correction methods are able to substantially account for skull variability or if the skull samples were sufficiently similar. In addition to inter-skull heterogeneity, there are two factors that further complicate the comparison of these results: transducer frequency and transducer coverage. Marsac et al. used a 1 MHz Imasonic 512 element phased array transducer, whereas Maimbourg et al. used a 900 kHz Supersonic Imagine 512 element phase array transducer. Higher frequencies are more challenging to phase correct because of their shorter periods. Thus, we may expect the FDTD and PSTD methods from those studies to improve their performance when used at the 670 kHz frequency of this study. However, at the same time, the transducer coverage in both those studies included only the superior portion of the skull. Increasing skull coverage would result in a larger range of incidence angles, which could potentially increase the difficulty of phase correction. These two effects act in opposite directions, and the strengths of their effects are not well known. However, from a preliminary comparison of these studies, we may expect the performances of the FDTD, PSTD, and HAS methods to be similar. It is ultimately challenging to compare results across studies because the experimental setups, skulls, CT parameters, and acoustic property relationships used are different. This motivates the need for an open access database^39^ for which different phase correction methods may be benchmarked against one another.

The results from this study further suggest that skull density ratio (SDR) may not be an ideal metric for predicting treatment efficiency. SDR is a selection criterion used by InSightec to determine a skull’s ease of treatment. Typically, the higher the SDR of a skull, the less power is needed to reach a threshold temperature rise at the focal spot. Although skulls A and B had very similar SDRs of 0.54 and 0.53, 21% more pressure (46% more intensity) could be transmitted through skull A than through skull B using the hydrophone correction method (Fig. 3a,b). The InSightec ray tracing results were also substantially different: 45% more target pressure and 48% more peak pressure (111% and 119% more target and peak intensities) could be transmitted through skull A than through skull B. Furthermore, skull C had the highest SDR of the three skulls but did not receive the highest focal spot pressure with any of the phase correction methods. Although there exists a statistically significant relationship between SDR and treatment efficiency^40^, the results from this study and from other studies^40,41^ provide evidence that SDR may be an imprecise predictor of treatment efficiency. Considered altogether, these data suggest that SDR may not fully encapsulate the complexity of skull aberrations.

### Analysis of phase correction efficacy

Dividing the transducer into subsets of elements helped identify areas in which each phase correction method could improve. In general, the anterior and temporal parts of the skull caused the most aberrations and were the most difficult to correct for. Figure 4b shows that sections 5 and 6 associated with the anterior skull each transmitted less than 60% hydrophone-corrected pressure to the target compared to the other sections. These two sections were also associated with the largest mean absolute phase errors (Fig. 4c). When no phase corrections were applied, these sections contributed negligible or negative pressure to the target, displaying the extent of aberrations caused by the skull. Similar observations were made regarding the temporal skull, which was associated with sections 4 and 7. The difficulties with treating through the anterior and temporal skulls have been observed during clinical treatments, in which treatment through the anterior skull is often avoided, and in some cases the elements interfacing with the temporal bone are deactivated as well. Deactivating elements in these sections would reduce the overall pressure loss compared to the hydrophone-corrected case, though doing so may cause the focal spot to become oblique. Furthermore, to achieve the desired temperature rise at the focal spot while using fewer elements, the transducer may need to be operated at higher powers. At higher powers, there is a larger risk of cavitation and element crosstalk, which may reduce patient safety and reduce the effectiveness of phase corrections, respectively. Overall, addressing these two regions of the skull may be where the most improvement can be gained.

Large phase errors are not necessarily more destructive than small phase errors. They are destructive when paired with large pressure amplitudes, but less destructive when paired with small pressure amplitudes. Figure 5f shows that on average, elements with larger HAS phase error were associated with lower pressure transmission through the skull. Less transmission through the skull suggests that the associated regions of skull were highly complex and aberrating, which may be why the HAS method was unable to model this complexity. When evaluating phase correction methods, phase error in itself can be a useful metric, though augmenting it with additional information such as pressure amplitude can provide a more holistic view.

### Study limitations

Although the performance of the HAS method is promising, it should be considered with the limitations of this study. First, experiments were performed on only three skulls, a small sample size that may not be representative of the entire patient population. Investigation into additional skulls will need to be performed, ideally spanning a larger range of SDR values. Second, only the geometric focus of the transducer was used as the target for phase correction. Although this may be sufficient for treatments requiring a small window of treatment, further investigation with electronic steering will be needed. Full characterization of the strengths and weaknesses of these methods will help identify the situations for which each method is suitable. Third, the positioning of the ex vivo skulls in these experiments did not fully replicate the positioning of patient skulls in clinical treatments. During essential tremor treatments, patient skulls are positioned off-center within the transducer to target the ventral intermediate nucleus of the thalamus^1^, the pallidothalamic tract^3^, or the subthalamic nucleus^4^. In contrast, the skulls in this study were centered within the transducer. Consequently, the incident angles of ultrasound on the skull may be less pronounced, potentially making it easier to focus through the skull without phase corrections. Setting the skull off-center is likely to reduce the recovered intensity for all phase correction methods. However, it is unclear how the no-correction-to-phase-correction improvement will change. Fourth, the HAS method’s current simulation time is not yet practical for a clinical setting. To seamlessly integrate the method into the current clinical workflow, simulation times that are less than one minute are desired^33^. One way to reduce simulation time is to parallelize computations using more computational resources. With complete parallelization of all elements, simulations may be performed in approximately 10 s. These study limitations motivate important future work to acquire additional data and to characterize the effectiveness of different phase correction methods.

## Conclusion

Compared to the standard of care InSightec ray tracing method, the hybrid angular spectrum method recovered more focal spot intensity at the target and resulted in less focal spot positioning errors. Using improved phase corrections to refocus the focal spot has great relevance for transcranial focused ultrasound ablation treatments. The improved targeting provides promise in improving treatment outcomes and streamlining the clinical workflow. It may also enable a larger subset of the patient population to receive treatment, and thus indirectly influence the patient screening process. These improvements are also highly relevant for applications in neuromodulation and blood brain barrier opening, which are currently being translated for human use.

## Methods

### Experimental setup

Three ex vivo human skulls were used in this study (Fig. 1a). The protocol was approved by the Virginia State Anatomical Program and all research was conducted in accordance with the guidelines specified by the Virginia State Anatomical Program, the University of Virginia medical research protocols, and the University of Virginia Institutional Review Board for Health Sciences Research. Informed consent to use the donors’ skulls for scientific research were obtained from the donors and donors’ next of kin.

The skulls were each fixed to a head frame prior to experimentation to ensure reproducible positioning between experiments (Fig. 1b). Computed tomography (CT) scans for all three skulls were acquired using CT parameters approved by the InSightec patient screening imaging protocol (Table 2). The skull density ratios (SDR) are also reported. In general, SDR is a measure of bone composition homogeneity on a scale of 0 to 1, with higher SDR associated with higher homogeneity. Prior to imaging or other experimentation, the skulls were degassed overnight with an Abbess Instruments acrylic vacuum chamber.

**Table 2:** CT parameters used for imaging the three ex vivo skulls.

Skull	Vendor	Tube voltage (kVp)	Reconstruction kernel	Compensation filter	Resolution	Skull density ratio
A	GE	120	BONEPLUS	Medium	0.5664 × 0.5664 × 0.625 mm	0.54
B	GE	120	BONEPLUS	Medium	0.5605 × 0.5605 × 0.625 mm	0.53
C	GE	120	BONEPLUS	Medium	0.5469 × 0.5469 × 0.625 mm	0.74

The skull and head frame were secured in a 670 kHz InSightec Exablate 4000 hemispherical phased array transducer and positioned in the clinical orientation; the anterior portion of the skull was closest to the anterior portion of the transducer. An Onda HNA-0400 needle hydrophone was mounted on an Onda 3-axis positioner system and used to perform 3D raster scans of the beam profile.

Prior to skull sonication, the position of the geometric focus in water was determined using a series of 2D raster scans. All transducer elements were turned on, and the focal spot position was localized by alternating between axial, coronal, and sagittal scan planes and centering the system on the position with maximum pressure. A three-plane cross section of the focal spot in water is shown in Supplementary Figure 8.

The InSightec workstation was operated in research mode and was supplied phase corrections prior to each sonication. All elements were fired simultaneously while the hydrophone was rastered in a 5 × 5 × 5 mm field of view grid. This 3D grid was acquired with 21 individual 5 × 5 mm sagittal scans with in-plane and through-plane step sizes of 0.25 mm. For each skull, data for all 21 sagittal scans and for all phase correction methods were acquired on the same day. The pulsing scheme was 2 ms on, 50 ms off using 20 W of electrical power. An Agilent DS07012B oscilloscope and the Soniq software from Onda were used to record the peak to peak voltages, which were then converted into pressure and intensity amplitudes.

### Skull registration

To accurately model the experimental setup, the position and orientation of the skull relative to the transducer was determined. Registration between the skull and head frame was not necessary, because their relative positioning and orientation were captured in the CT images. The head frame was fabricated according to computer aided design (CAD) specifications, in which the position of the head frame relative to the transducer was also specified. Eight 2 mm diameter tantalum beads were epoxied onto the head frame at specified locations denoted in the CAD. These beads were installed prior to CT imaging and served as fiducial markers to register the CT images to the transducer (Supplementary Figure 10). The centroid of each bead was used as its position in CT space. A singular value decomposition-based least squares registration^42,43^ was performed to achieve point-wise registration between fiducial marker positions in CT and transducer space. The transformation matrix that registers the two coordinate systems is:

1 $${P}_{transducer}=R{P}_{CT}+Tr$$

where P_transducer_ is a 3 × 8 matrix of fiducial marker positions in transducer space, P_CT_ is a 3 × 8 matrix of fiducial marker positions in CT space, R is a 3 × 3 rotation matrix, and Tr is a 3 × 8 translation matrix. The rotation matrix R is calculated as

2 $$R=U{V}^{T}$$

where U and V are the unitary matrices from the singular value decomposition of the matrix H

3 $$SVD(H)=U\Lambda {V}^{T}$$

4 $$H=\left({P}_{CT}-\frac{1}{8}\sum_{i=1}^{8}{p}_{CT,i}\right){\left({P}_{transducer}-\frac{1}{8}\sum_{i=1}^{8}{p}_{transducer,i}\right)}^{T}$$

and where p_CT,i_ is a 3 × 1 position vector of the ith fiducial marker in CT space, p_transducer,i_ is a 3 × 1 position vector of the ith fiducial marker in transducer space, and T is the transpose operator. With values for P_CT_, P_tranducer_, and R, the translation matrix Tr can then be calculated using Eq. (1). The resulting transformation matrix was used to resample the CT images into transducer space to create a set of registered CT images. Axial slices with 0.5 × 0.5 × 1 mm resolution were used for the straight ray tracing, InSightec ray tracing, and HAS phase correction methods. After registration, fiducial marker centroid positions were 0.20 ± 0.07 mm away from their intended positions.

### Phase correction methods

To target the geometric focus of the transducer, four different methods were used to estimate phase corrections. These methods were (i) straight ray tracing, (ii) InSightec ray tracing, (iii) HAS, and (iv) hydrophone. The straight ray tracing, InSightec ray tracing, and HAS methods require computational models of the skulls’ acoustic properties in order to compute phase corrections. These skull models were generated using CT images of the skulls, which were first registered to the transducer as described above. Each set of images represented a 3D volume containing the skull, and for each voxel in the volume, properties of acoustic velocity, density, and attenuation were assigned based on its Hounsfield unit (HU). The InSightec ray tracing method used a proprietary algorithm to map HU to acoustic properties, whereas the straight ray tracing and HAS methods used an acoustic velocity relationship from Webb et al.^44^ and an attenuation relationship from Leung et al.^38^ to map HU to acoustic properties. All simulations were performed in transducer space. Computations were performed on an Intel Core i7-8700K 3.7 GHz CPU processor.

### Straight ray tracing method

A straight ray tracing method was used to benchmark against the proprietary InSightec ray tracing method. A ray was drawn from each transducer element to the geometric focus of the transducer. These rays were divided into 0.05 mm segments and were used to resample the skull model. The traversal time within each segment was summed along the ray to yield the total arrival time. Time delays were calculated based on relative arrival times, and these time delays were in turn used to calculate phase corrections for a 670 kHz signal. Simulations for each element required 1.51 ± 0.06 ms to compute; 1024 total simulations required approximately 2 s. Computation time can be reduced by using lower resolutions (Supplementary Figure 11).

### InSightec ray tracing method

Although the InSightec ray tracing method remains proprietary, we were able to calculate phase corrections by using the InSightec workstation in clinical mode. After uploading the registered CT images, we followed the clinical workflow for treatment planning (Supplementary Figure 12). Because the skull was already registered to the transducer in these images, no further registration was necessary. The phase corrections could be found in the system logs.

### HAS method

The HAS method was implemented as previously described^38^. Briefly, the pressure plane from each element was propagated through the skull model until it reached a plane containing the geometric focus. The phase at the geometric focus was then saved. A field of view of 400 × 400 × 240 mm was used with a voxel size of 1 mm isotropic. For a center frequency of 670 kHz, a maximum voxel size of 1.5 mm isotropic is suggested (Supplementary Figure 13)^45^. Simulations for each element required 9.58 ± 1.98 s to compute; 1024 total simulations required approximately 30 min (using six parallel cores on the CPU processor).

### Hydrophone method

While the other methods require models of the skull to compute phase corrections, the hydrophone method directly accounts for skull aberrations using experimental data. Individual elements were fired sequentially, and their time series signals were measured with the hydrophone positioned at the geometric focus. The pulse duration used was 3 ms at 375 W electrical power, the sampling frequency was 15 MHz, and the sampling duration was 7 ms. Data were acquired for all 1024 transducer elements using a TiePie Handyscope HS6 Diff and a Panametrics model 5676 preamplifier. The recorded signal from each individual element was transformed to the frequency domain using a fast Fourier transform, and the coefficient of the main frequency component was used to determine the phase of the signal.

### Validation of simulations in water

To confirm accurate transducer modeling, we performed sonications in water and applied phase corrections calculated with each method. Due to variability in the transducer fabrication process, transducer elements are not located exactly on a sphere, and baseline phase corrections are needed to target the nominal geometric focus. With no geometric correction, the pressure at the target is high, though it is improved after using hydrophone method phase corrections (Supplementary Figure 9). The straight ray tracing, InSightec ray tracing, and HAS method phase corrections also improved upon the no correction case by accounting for element positions. The resulting pressures between the three methods were very similar, showing that the three methods were modeling the transducer similarly. The hydrophone method performed better than the other three methods because it can robustly account for variability in transducer positioning.

### Quantitative metrics

Six quantitative metrics were used to evaluate phase correction performance. Focal spot pressures were indexed at the target position and at the position of peak pressure. Intensities were calculated and normalized to the results from the hydrophone phase correction method, the gold standard for recovering maximum intensity. This normalization removed the influence of skull-dependent attenuation and allowed phase correction performance to be compared across skulls. Positioning errors were calculated as the difference between the target position and the focal spot position, defined to be at the voxel of peak pressure. Focal spot volume was measured at pressure full width half maximum.

### Analysis of phase correction efficacy

To better understand sources of error in phase correction, subsets of elements were analyzed to determine their contributions to the target pressure. By the superposition principle, the net focal spot pressure is equivalent to the sum of pressures contributed by the individual elements. Therefore, the focal spot pressure can be computed using time series data acquired with the hydrophone method, detailed in the “Phase correction methods” section. Because the time series data were only acquired at the target position, only the target pressure could be computed for this analysis. The effective pressure contribution by any group of elements can be determined by subtracting the group’s signals from the overall sum, then measuring the change in pressure, as shown in the following expression

5 $${P}_{G}= P\left(\sum_{i=1}^{1024}{s}_{i}(t){ e}^{j{\phi }_{i}}\right)-P\left(\sum_{i=1}^{1024}{s}_{i}(t) {e}^{j{\phi }_{i}}-\sum_{g\in G}{s}_{g}(t){ e}^{j{\phi }_{g}}\right)$$

where P_G_ is the effective pressure contribution by a group G of elements, P() is the pressure averaged over 100 cycles, s_i_ is the time series data for element i, and φ_i_ is the phase correction for element i calculated with one of the methods. In the second term, the subtraction from the overall sum is necessary to elucidate destructive interference that persists after applying phase corrections. For example, suppose there are two groups of elements, A and B, and that elements within each group are perfectly in phase. Let us also assume that group A contributes nine times the pressure as group B. If the two groups were perfectly in phase with one another and summed, group B would contribute 1/10 of the focal spot pressure. However, if the two groups were offset in phase by π radians, their summation would result in less focal spot pressure than when using group A alone. Therefore, group B would have a detrimental effect to the focal spot pressure and would have a negative effective contribution. This analysis allows us to observe how one group of elements interacts with the remaining elements of the transducer.

Two types of element groupings were investigated: (i) grouping by physical location of transducer elements and (ii) grouping by phase error. Mean absolute phase errors were also calculated using the following expression

6 $$Mean \; absolute \; phase  \; error= \frac{1}{n}\sum_{i=1}^{n}abs({\varphi }_{hydrophone,i}-{\varphi }_{method,i})$$

where n is the number of elements in the subset, φ_hydrophone,i_ is the phase for element i calculated with the hydrophone method, and φ_method,i_ is the phase for element i calculated with one of the other methods.

## Supplementary Information

Supplementary Information.
