# Supplementary material for: Transcranial focused ultrasound phase correction using the hybrid angular spectrum method
Source: Sci Rep. 2021 Mar 22;11:6532. doi: 10.1038/s41598-021-85535-5 (PMC7985511; doi:10.1038/s41598-021-85535-5)
Supplement: Supplementary file 1 — Supplementary Information. [file 41598_2021_85535_MOESM1_ESM.docx]

Supplementary information to:

Transcranial focused ultrasound phase correction using the hybrid angular spectrum method

Steven A. Leung^1,*^, David Moore^2^, Taylor D. Webb^3^, John Snell^2,4^, Pejman Ghanouni^5^, Kim Butts Pauly^1,3,5^

^1^ Department of Bioengineering, Stanford University, Stanford, California, USA

^2^ Focused Ultrasound Foundation

^3^ Department of Electrical Engineering, Stanford University, Stanford, California, USA

^4^ Department of Neurological Surgery, University of Virginia, Charlottesville, Virginia, USA

^5^ Department of Radiology, Stanford University, Stanford, California, USA

^*^ Corresponding author (stevenleung@stanford.edu)

**Skull A**


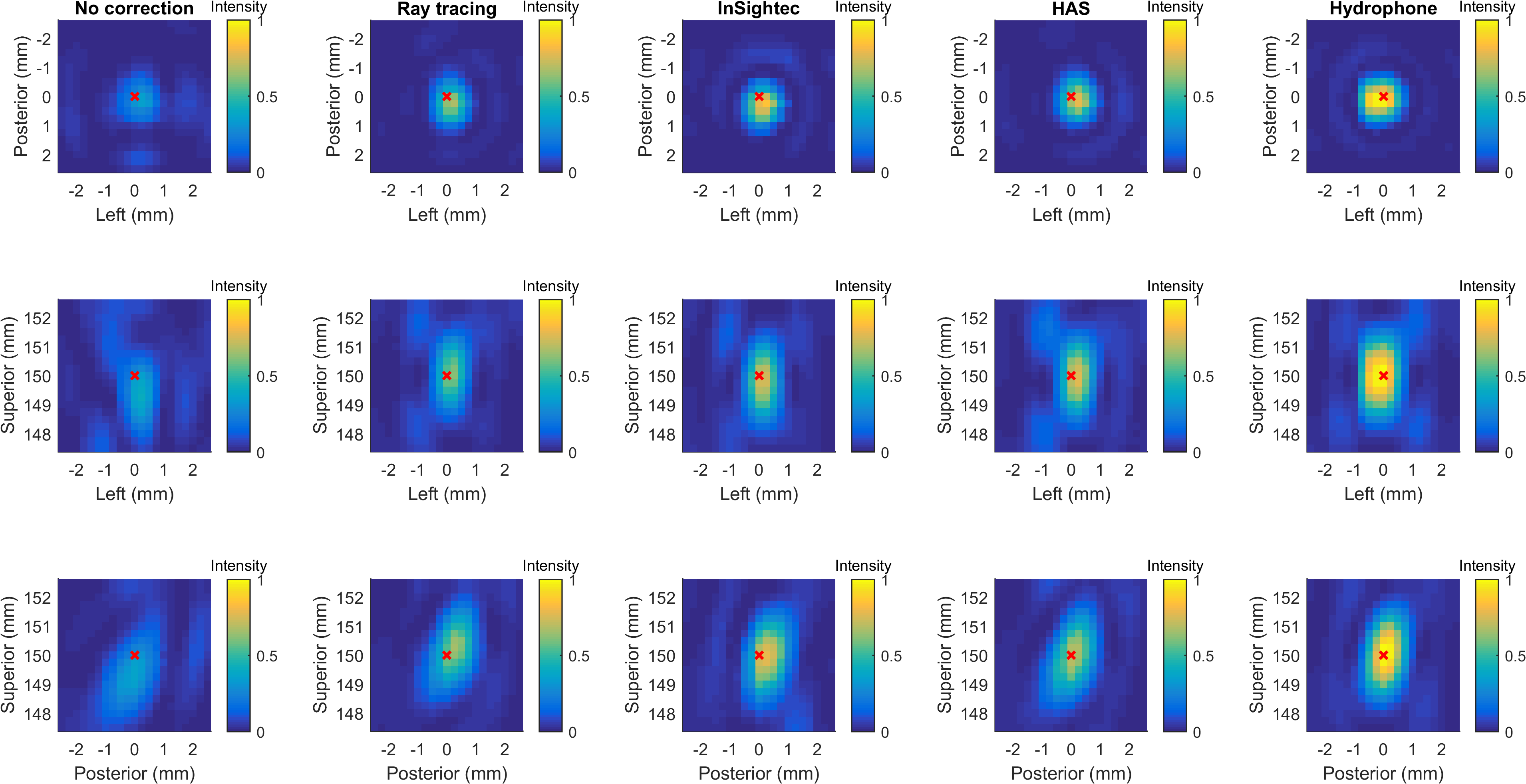


**Supplementary Figure 1.** Three-plane cross sections of the focal spots generated with each phase correction method (Skull A). Corresponds with Figure 2 in the main text. The red x marks the location of the targeted position.

**Skull B**


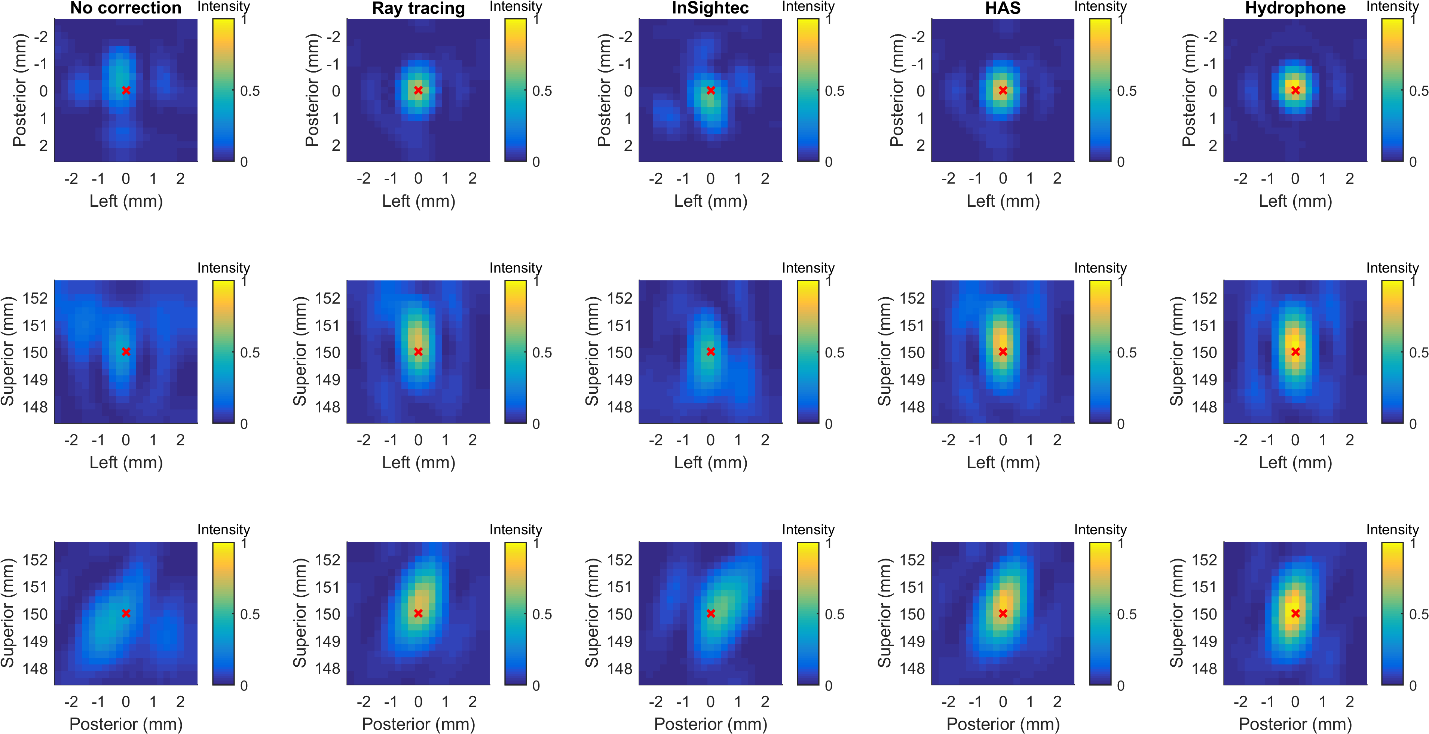


**Supplementary Figure 2.** Three-plane cross sections of the focal spots generated with each phase correction method (Skull B). Corresponds with Figure 2 in the main text. The red x marks the location of the targeted position.

**Skull C**


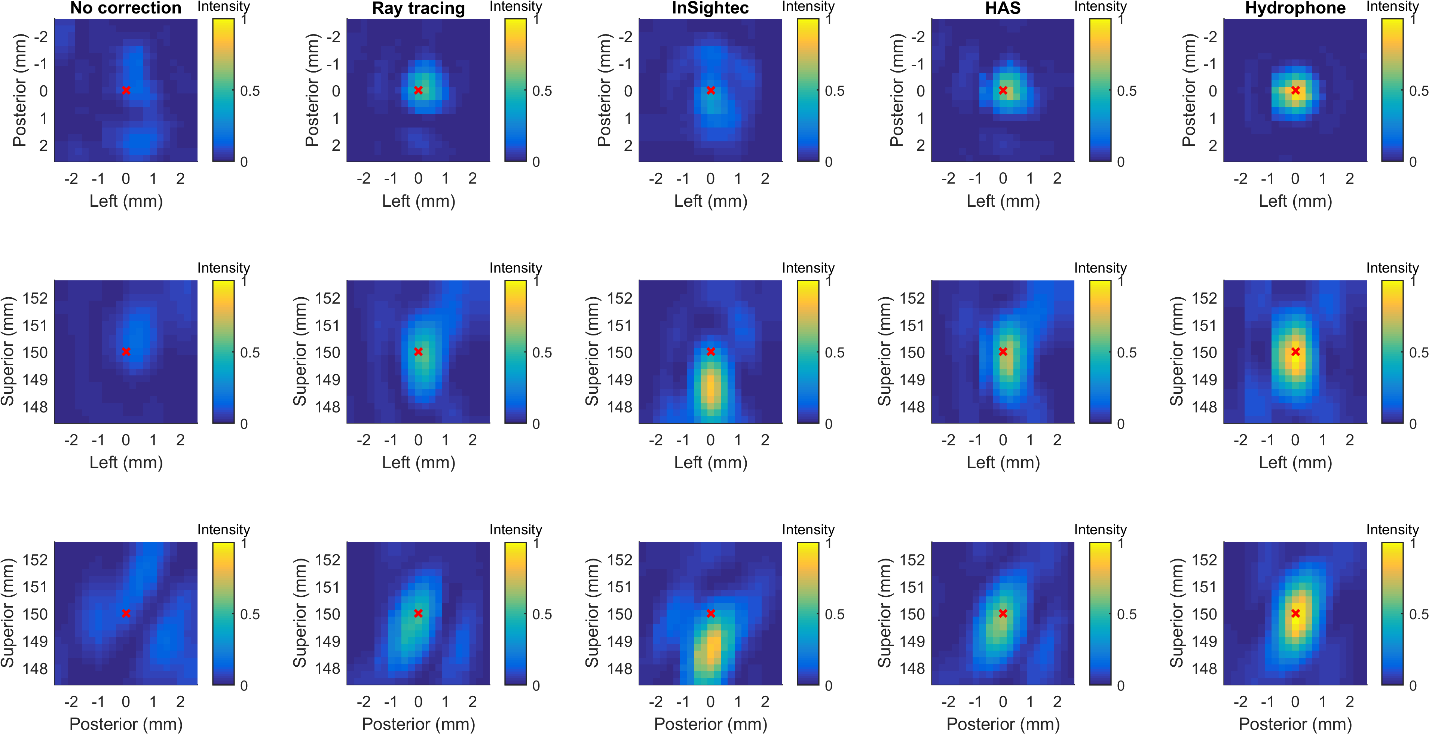


**Supplementary Figure 3.** Three-plane cross sections of the focal spots generated with each phase correction method (Skull C). Corresponds with Figure 2 in the main text. The red x marks the location of the targeted position.

**Skull A**


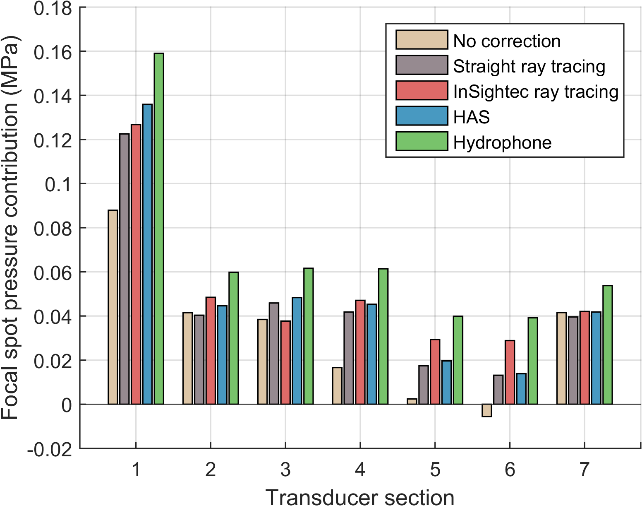

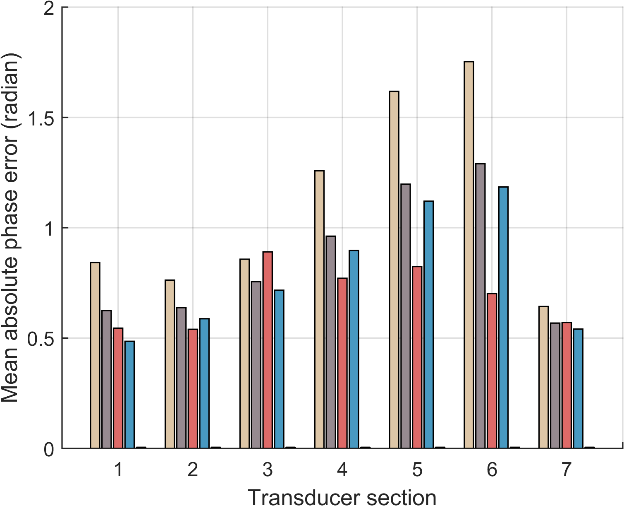


a) b)

**Supplementary Figure 4.** Analysis of phase correction efficacy for each transducer section (Skull A). Corresponds with Figure 4 in the main text. a) Contributions by each transducer section to target pressure, calculated with Equation 5. Negative values denote transducer sections whose net ultrasound signal was out of phase with the rest of the transducer. b) Mean absolute phase errors for each transducer section.

**Skull B**


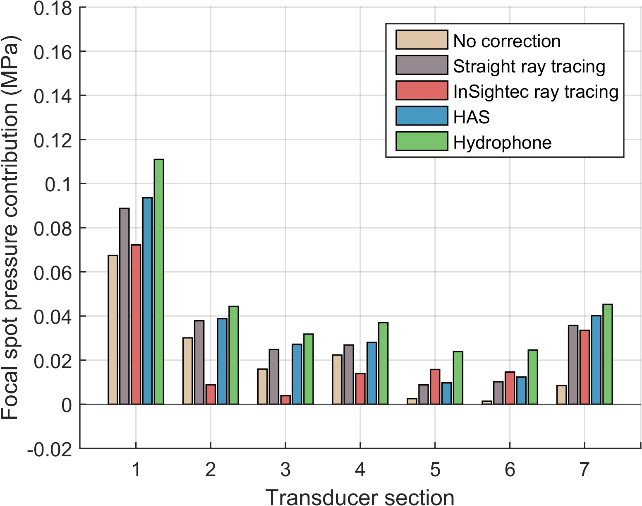

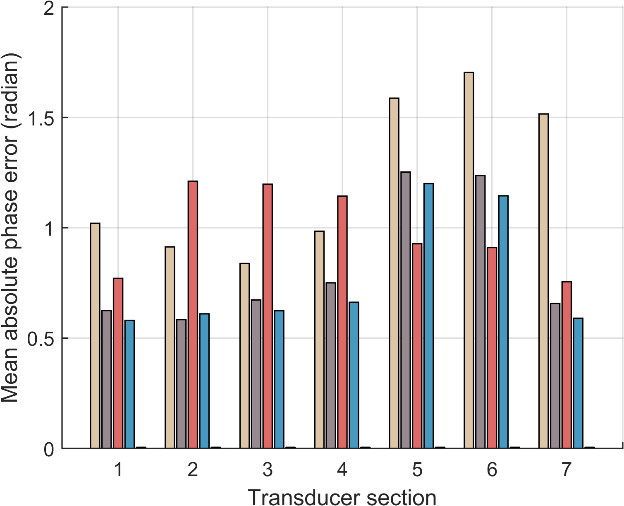


a) b)

**Supplementary Figure 5.** Analysis of phase correction efficacy for each transducer section (Skull B). Corresponds with Figure 4 in the main text. a) Contributions by each transducer section to target pressure, calculated with Equation 5. Negative values denote transducer sections whose net ultrasound signal was out of phase with the rest of the transducer. b) Mean absolute phase errors for each transducer section.


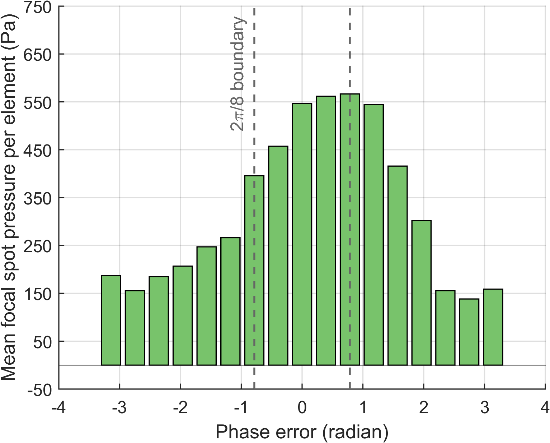

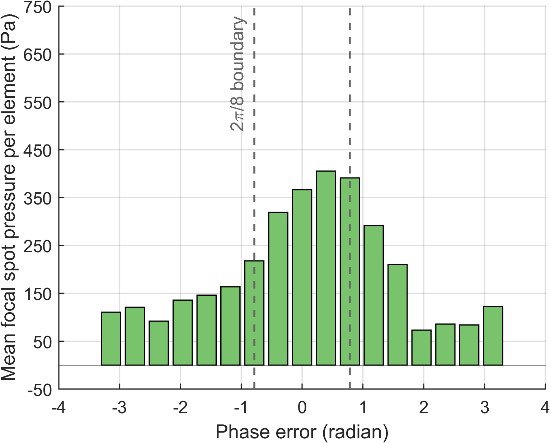


a) b)

**Supplementary Figure 6.** Mean pressure contributed per element. Subsets of elements are grouped by HAS phase error. Corresponds with Figure 5 in the main text. Values are shown for a) Skull A and b) Skull B.


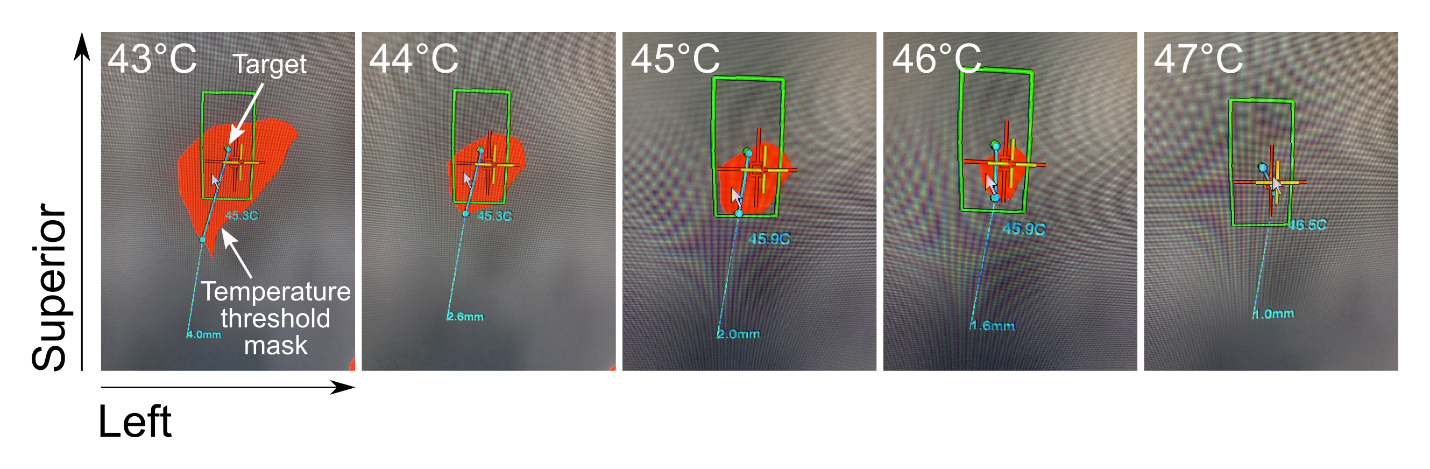


**Supplementary Figure 7.** Observational data showing an inferior-shifted initial focal spot for a patient with high SDR (0.77). Coronal images are shown for the initial focal spot (sonication 2, 3000 J, 10 s) with the MR thermometry phase encode direction along the superior-inferior axis. The patient was treated for essential tremor and the InSightec ray tracing method was used to calculate phase corrections. The red binary mask shows the region for which the tissue temperature exceeded a given temperature threshold, denoted in the top left of each image. The green dot marks the targeted position, and the focal spot can be seen to be displaced inferior to the target. The blue line is drawn from the targeted position to the bottom edge of the red binary mask, and that distance is labeled in blue. The red crosshair is centered on the pixel with the highest temperature, and the yellow crosshair is user-controlled to generate the temperature versus time curve. The blue text below the green rectangle reports the temperature at the cursor.


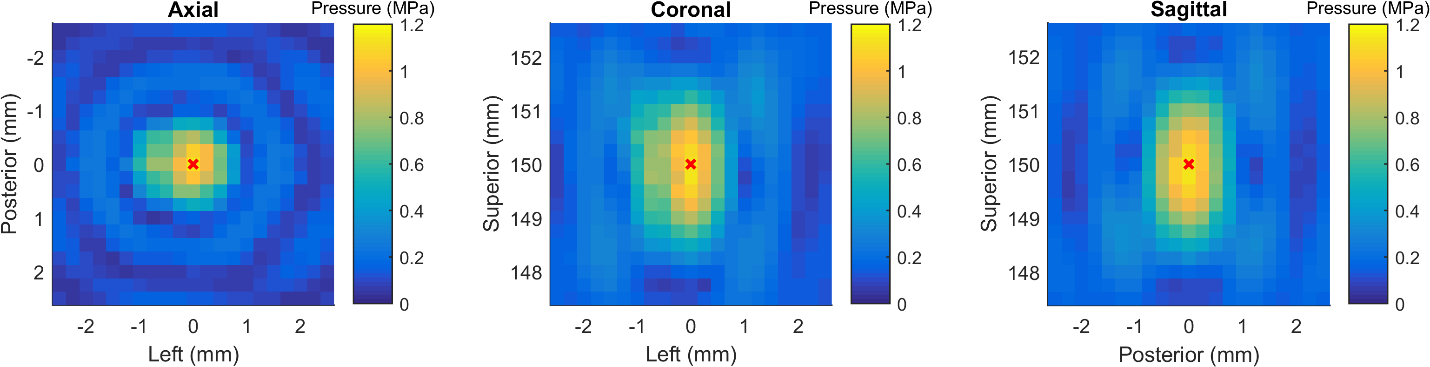


**Supplementary Figure 8.** Three-plane cross section of the focal spot in water using 20 W of electrical power. The red x marks the location of the geometric focus, which was earlier determined using a series of 2D scans to localize the focal spot. In this sonication mode, the InSightec transducer automatically applies phase corrections to account for element positioning.


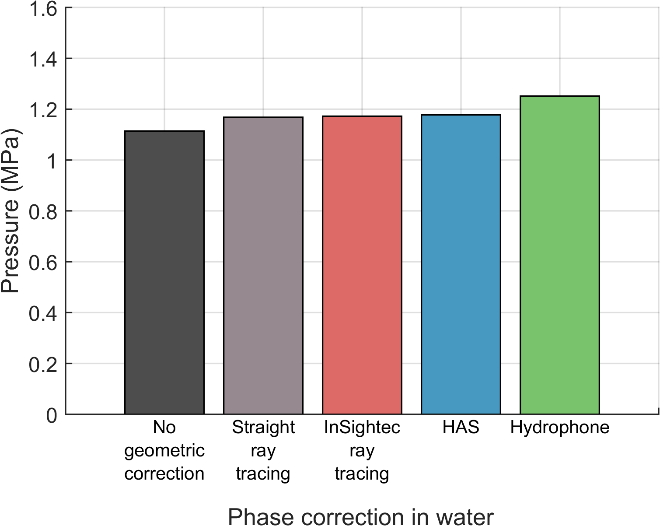


**Supplementary Figure 9.** Validation of phase correction in water. The nominal geometric focus was targeted using 20 W of electrical power. Because the elements are not located exactly on a sphere, the straight ray tracing, InSightec ray tracing, and HAS methods were used to calculate phase corrections to account for element positioning. Element positions were referenced from an InSightec transducer geometry file. The resulting pressures between the three methods were very similar, showing that the three methods were modeling the transducer similarly. The hydrophone method performed better than the other three methods because it can robustly account for variability in transducer positioning.


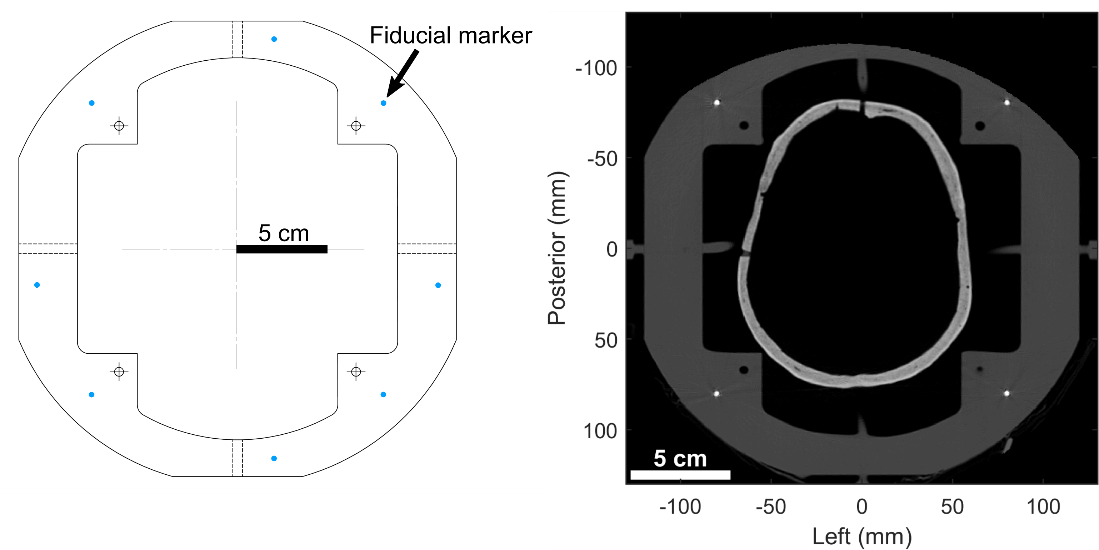


**Supplementary Figure 10.** Registration of CT coordinate space to transducer coordinate space. A singular value decomposition-based least squares registration ^[42,43]^ was performed to achieve point-wise registration between CT and transducer positions (Equation 1). Tantalum bead fiducial markers were used for registration. The relative positioning between head frame and transducer are specified in the computer aided design (CAD) file. After performing this registration step, the skull, head frame, and transducer are registered. On the right plot, only four tantalum beads can be seen because the other four are in a different plane.


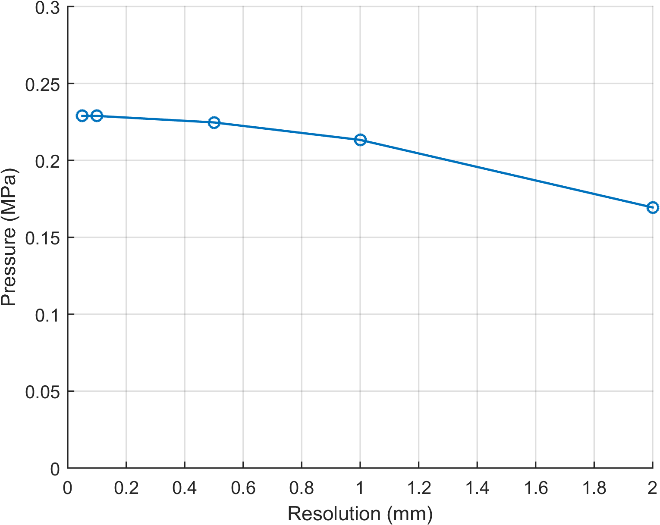

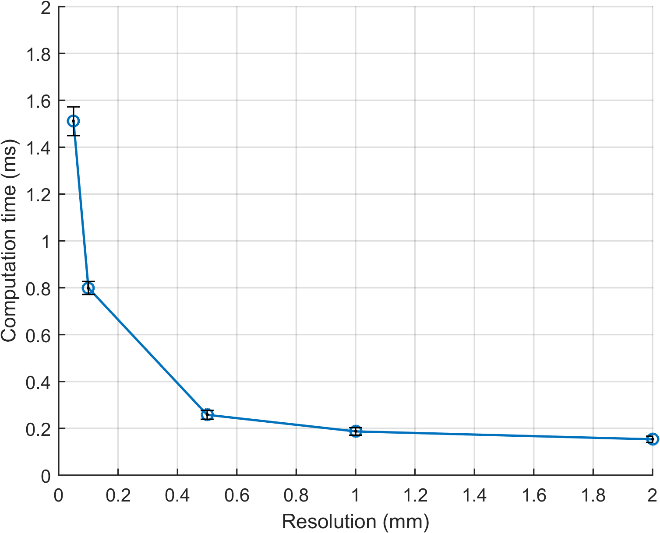


1. b)

**Supplementary Figure 11.** Performance of the straight ray tracing method as a function of resolution (Skull C). a) Pressure at the target. b) Computation time for a single element. Error bars show the standard deviation resulting from 50 repetitions.


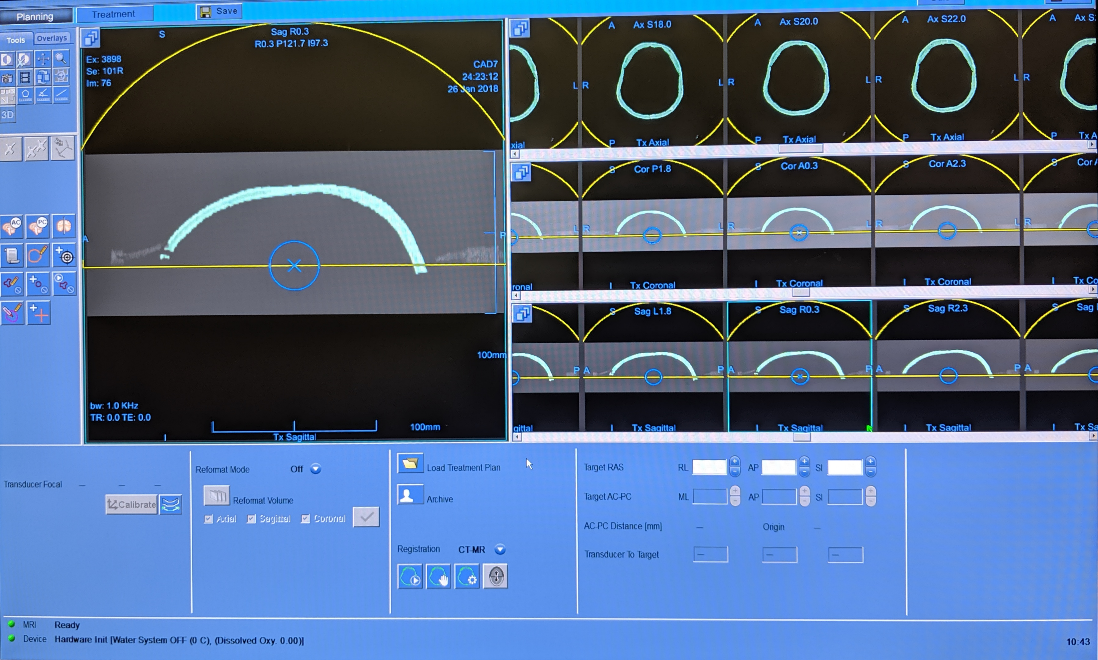


**Supplementary Figure 12.** Screenshot of the InSightec workstation operating in clinical mode. The position of the transducer (yellow outline) and target (blue x) are shown relative to the registered skull. For illustration, the image window and level have been adjusted to show the head frame. When calculating phase corrections, a different set of images with the head frame segmented away is used.


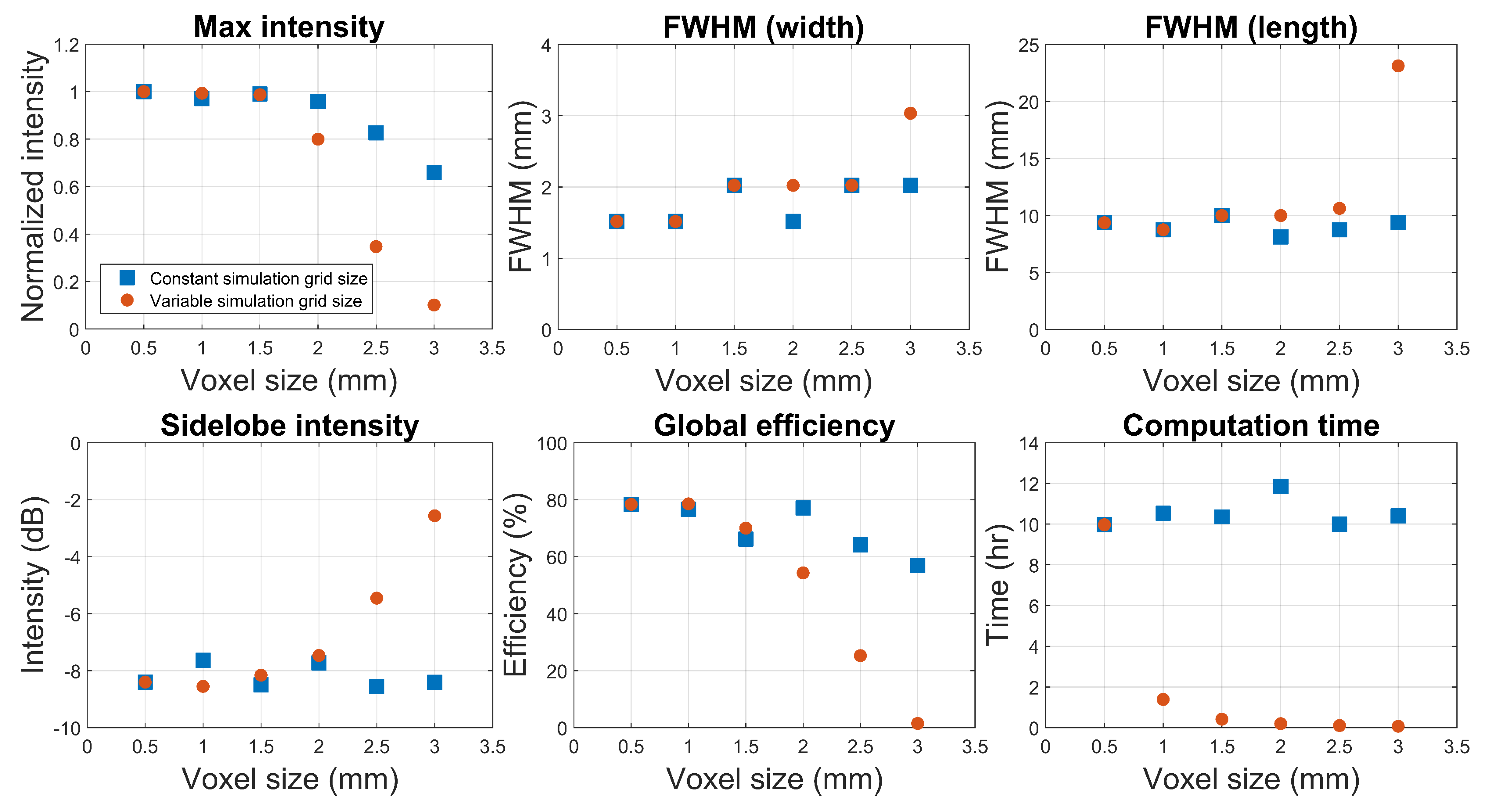


**Supplementary Figure 13.** Metrics used to characterize trade-offs between skull model voxel size and characteristics of the refocused beam when using the HAS method. A skull model with a native voxel size of 0.5 mm was downsampled to larger voxel sizes. Constant simulation grid size: simulation voxel size was maintained at 0.5 mm. Lower resolution skull models were upsampled to match the resolution of the simulation. Variable resolution grid size: simulation voxel size was variable between 0.5 mm to 3 mm, each time matching the voxel size of the skull model. Figure reproduced here with permission ^[45]^.
